# Holmium Laser Enucleation of the Prostate: Modified Two-Lobe Technique versus Traditional Three-Lobe Technique—A Randomized Study

**DOI:** 10.1155/2019/3875418

**Published:** 2019-09-30

**Authors:** Congcong Xu, Zhen Xu, Caixiu Lin, Sheng Feng, Mingwei Sun, Jijun Chen, Yichun Zheng

**Affiliations:** ^1^Department of Urology, The Second Affiliated Hospital of Zhejiang University School of Medicine, Hangzhou, Zhejiang, China; ^2^Department of Neurology, The Second Affiliated Hospital of Zhejiang University School of Medicine, Hangzhou, Zhejiang, China; ^3^Department of Urology, The First Affiliated Hospital of Zhejiang Chinese Medical University, Hangzhou, Zhejiang, China; ^4^The Children Hospital of Zhejiang University School of Medicine, Hangzhou, Zhejiang, China

## Abstract

**Background:**

Holmium laser enucleation of the prostate (HoLEP) is considered the standard endoscopic treatment of benign prostatic hyperplasia (BPH), but traditional HoLEP surgery will cause some postoperative complications. This study was attempted to evaluate the safety and efficacy of modified two-lobe technique versus traditional three-lobe technique of HoLEP focusing mainly on incidences of retrograde ejaculation (RE) and urinary incontinence (UI).

**Methods:**

From March 2014 to February 2017, 191 men with BPH were randomly assigned to two groups: 97 underwent modified two-lobe technique; 94 underwent traditional three-lobe technique. All patients were followed up for 12 months. Primary outcomes were incidences of RE and UI, and secondary outcomes were international prostate symptom score (IPSS), quality of life (QOL), maximal urine flowing rate (MFR), and residual urine among the studied patients.

**Results:**

Compared with the traditional technique, patients in the modified group had a statistically significant decrease in frequency of UI (1.03% vs 8.51%, *p*=0.036) and RE in the 6^th^ month (33.33% vs 63.64%, *p*=0.030) and 12^th^ month (13.33% vs 50%, *p*=0.034) and a significant increase in ejaculatory volume in the 6^th^ month (*p*=0.050) and 12^th^ month (*p*=0.003). Besides, the modified HoLEP was more beneficial to patients according to the change of QoL score at 1 month (*p*=0.002), 3 months (*p*=0.004), 6 months (*p*=0.026), and 12 months (*p*=0.015).

**Conclusions:**

The modified two-lobe technology of HoLEP reduced the incidence of RE and UI, which improved the quality of life of the patients after surgery compared to the traditional three-lobe technology. This trial is registered with ChiCTR1800018553.

## 1. Introduction

Benign prostatic hyperplasia (BPH) is a common medical condition in elderly men, and although it can be managed with medication, surgery remains the mainstay of treatment. Nowadays, many studies have demonstrated the advantages of HoLEP in resecting a larger amount of tissue compared to transurethral resection of the prostate (TURP) and photovaporization of the prostate (PVP) [1–3].

Nevertheless, long-term data indicated that enucleation could cause postoperative complications, including retrograde ejaculation (RE) and urinary incontinence (UI) [4, 5]. Shigemura et al. reported that among 497 patients who had been treated by 39 surgeons, the incidence of UI was >10% [6]. A recent study reported a 73.1% rate of RE following HoLEP [7]. Urologists thought that patients' RE or UI after HOLEP was probably because of an excessive damage to bladder neck and other normal tissue during surgery [8, 9]. We tried to introduce a set of modified techniques of HoLEP to reduce the incidences of RE and UI on the basis of anatomy and physiology [10, 11].

## 2. Methods

### 2.1. Study Design

Inclusion criteria were as follows: patients aged >50 years, refractory LUTS secondary to BPH, International Prostate Symptom Score (IPSS) of >15, maximum urinary flow rate (*Q*_max_) of <15 ml/s or patients with acute urinary retention secondary to BPH, whose trial of voiding had failed, and prostate size on preoperative transrectal ultrasonography (TRUS) was 40–150 ml. Exclusion criteria included history of previous prostate or urethral surgery and voiding disorders not related to benign prostatic hyperplasia. If suspected, prostate carcinoma was ruled out by biopsy.

**Figure 1:**
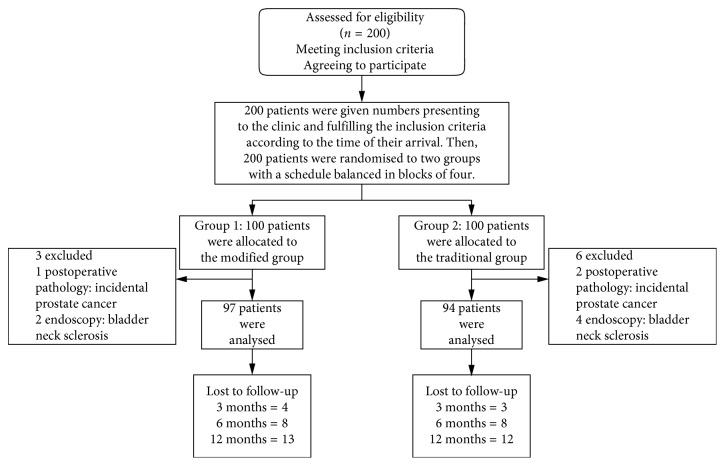
Flow of participants through the study.

Two hundred patients who met the inclusion criteria with BPH were recruited from March 2014 to February 2017. Patients participating in the study were randomly divided into 2 groups (using consecutively numbered envelopes containing the treatment applied). In the course of the experiments, 3 and 6 patients were excluded in the modified and traditional groups, respectively (Figure 1). The patient's age, prostate volume, prostate-specific antigen (PSA), postvoid residual urine volume (PVR), *Q*_max_, IPSS, Qol score, and International Index Of Erectile Function (IIEF) were recorded before operations. Perioperative outcomes, like operative time, transfusion rate, catheter duration, hospital stay, and drop in HGB levels were also collected. After surgery, follow-up was scheduled by the investigator who was blinded to the treatment grouping at 1, 3, 6, and 12 months. The follow-up protocol included checking for PVR, IPSS, QoL score, *Q*_max_ questionnaires, IIEF score, UI that was diagnosed by a thorough history and pad use, urinary retention, ejaculatory volume and RE that was evaluated by semen analysis, and postejaculatory urinalysis [12]. In case of a significant deterioration in the micturition parameters, further investigations were conducted and repeat surgeries were performed when indicated.

### 2.2. Surgical Procedures

All surgical procedures were performed under general anesthesia by a single surgeon with 8 years of experience in HoLEP, who have performed 500 HoLEP procedures. Holmium: YAG laser (fiber size 550 *μ*m; Versa Pulse Select, Coherent Corp., Palo Alto, Calif.) with a power setting of 2 J/40–50 Hz and a 26-F Olympus continuous fluid irrigation resectoscope with 0.9% saline as the irrigation fluid were used for HoLEP. At the end of the surgery, a 20-F three-way catheter was inserted and retained in situ until the urine was clear.

#### 2.2.1. Traditional HoLEP

HoLEP was performed as per the procedure previously mentioned by Tan and Gilling [13].

**Step 1 (enucleation of the lobes).**

Bilateral bladder neck incisions were made at the 5 and 7 o'clock positions, and the depth was increased until all the circular fibers had been divided. The incisions were extended downward just adjacent to the verumontanum. The distal ends of the bladder neck incisions were then joined just proximal to the verumontanum with a transverse incision, and the median lobe was dissected on the capsule in a retrograde fashion toward the bladder neck. Next, the lateral lobes were undermined on each side by extending the initial bladder neck incision laterally and circumferentially at the apex, working toward the 2 and 10 o'clock positions. The plane was developed from the apex toward the bladder neck. A bladder neck incision was also made at the 12 o'clock position, down to the capsule. A sweeping motion was used to continue the incision circumferentially, laterally, and distally, until the resectoscope could be partially withdrawn, and the upper and lower resection planes could be visualized and connected. Once each of the lateral lobes had been released from the bladder neck, hemostasis was performed with a defocused laser beam.

**Step 2 (morcellation of the prostate fragments).**

A mechanical morcellator extracted the tissue using reciprocating blades and a high-powered suction inside the bladder. Fragments of the tissue passed through the suction tubing assisted by using a roller pump and were collected in a special sock that fitted over the end of the tubing.

#### 2.2.2. Modified HoLEP

**Step 3 (enucleation of the lobes).**

**Figure 2:**
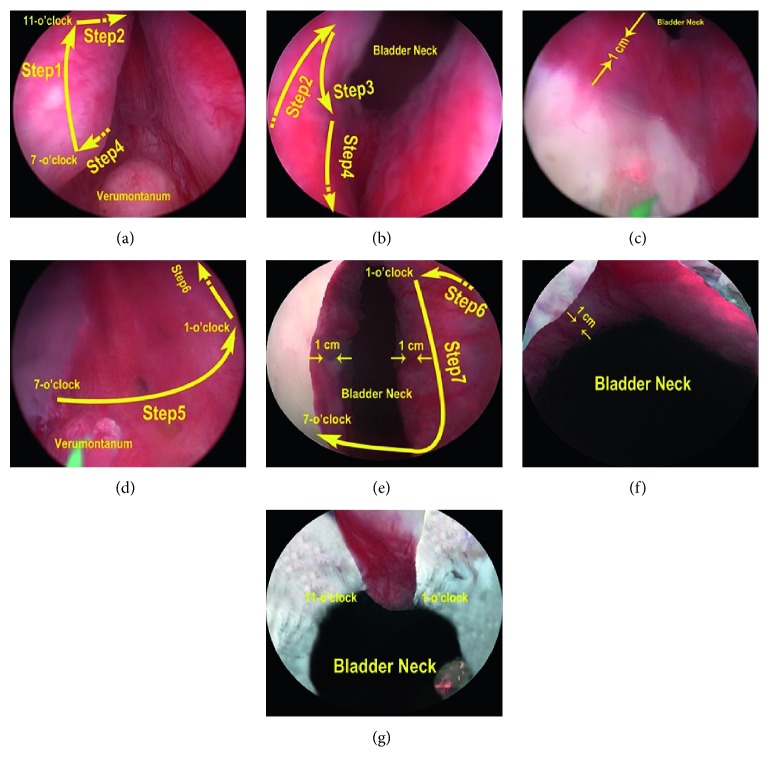
Surgical procedures of modified two-lobe technique. (a) Step 1: incision is made circumferentially from the 7 o'clock position to the 11 o'clock position near the verumontanum. (a, b) Step 2: incision is lengthened down to 11 o'clock, approximately 1 cm from the bladder neck. (b) Step 3: Incision is made laterally to the 7 o'clock position near the bladder neck. (a, b) Step 4: incision is made back to the starting point. (c) Bladder neck after the unilateral lobe has been released. (d) Step 5: incision is made circumferentially from the 7 o'clock position to the 1 o'clock position near the verumontanum. (d, e) Step 6: incision is lengthened down to 1 o'clock, approximately 1 cm from the bladder neck. (e) Step 7: incision is made back to the 7 o'clock near the verumontanum. (f) Bladder neck after three lobes has been released. (g) The mucous membranes of the prostatic urethra from the 11 o'clock to 1 o'clock positions are preserved.

The first incision was made circumferentially extending from the 7 o'clock position to the 11 o'clock position near the verumontanum, and the depth was extended into the surface of the glands (Figure 2 Step 1). Subsequently, the incisions were lengthened down to the 11 o'clock position, approximately 1 cm from the bladder neck (Figure 2 Step 2). A sweeping motion was used to continue the incision to the 7 o'clock position proximal to the bladder neck, laterally (Figure 2 Step 3). After this, the incisions were joined at the 7 o'clock position near the verumontanum (Figure 2 Step 4). Following the incisions, the lateral lobe was thoroughly enucleated. Once the unilateral lobe had been released, more space was available for enucleating the remaining lobes.

Then, the incision was made, commencing from the previous 7 o'clock position proximal to the verumontanum toward 1 o'clock position, and the depth was extended into the surface of glands (Figure 2 Step 5). Subsequently, at the 1 o'clock position, approximately 1 cm from the bladder neck, the incisions were extended inward (Figure 2 Step 6). A sweeping motion was used to continue the incision to the previous 7 o'clock position proximal to the bladder neck, laterally (Figure 2 Step 7). Following the incisions, the remaining lobes were thoroughly enucleated.

Caution was required during the surgery to avoid damaging the mucosa of the bladder neck and part of the membranous urethra, as well as the circular fibers of the internal urethral sphincter (Figure 2(f)). The mucous membrane of the prostatic urethra from the 11 o'clock to 1 o'clock positions were preserved as much as possible (Figure 2(g)). Also, the edge of some glands mostly overtopped the verumontanum; hence, to enucleate these glands completely, the surgeon had to pry them up. Some oversized glands needed to be excised to half their size, to make it easier for them to pass through the bladder neck; some glands, whose edge extended beyond the neck of bladder, swelled toward inside the bladder so that the enucleation could be performed along the edge of these glands in order to protect circular fibers.

**Step 4 (morcellation of the prostate fragments).**

Morcellation was performed as mentioned in Step 2 of Section 2.2.1. Before the end of the surgery, 20 mg of IV furosemide was routinely administered; then, a 22-F three-way Foley catheter was placed in the bladder and connected to a drainage bag.

### 2.3. Statistical Analysis

Continuous data were recorded as mean ± SD, if normally distributed, or as mean rank, if not normally distributed. Normally distributed data were analyzed with Student's *t* test, while the Wilcoxon rank sum test was used for data that were not normally distributed. Categorical data were compared with the chi-square test or Fisher's exact test (proportions). *p* values of <0.05 were considered statistically significant. Statistical analysis was performed using SPSS 23.0 for Windows (SPSS Inc, Chicago, Ill).

## 3. Results

As shown in Table 1, there were no statistically significant differences in the baseline characteristics between the two groups. IIEF scores were compared between the two groups among patients who had a sexual urge (30/97 in the modified group; 22/94 in the traditional group).

Preoperatively, there were no significant differences between the analyzed groups (Table 2).

Postoperative outcomes of the two groups are given in Table 3. Some patients discontinued postoperative assessments until 12 months of follow-up. After the surgery, obvious improvement of QoL score was observed in the modified group at 1, 3, 6, and 12 months, and this difference was found to be statistically significant. One month after the surgery, the change in maximum urinary flow rate (i.e., detrusor pressure) was significantly lower in the modified group than in the traditional group.

Table 4 shows the early and late postoperative complications. Early postoperative complications (at 1-month follow-up) were seen in 10 patients: 2 from the modified group and 8 from the traditional group. Transient UI was more frequent significantly in the traditional group (*p*=0.036). RE was the most common complication, especially in the traditional group. Among patients who had a sexual urge, the occurrence rate of RE was 33.33% in the modified HoLEP group and 63.64% in the traditional HoLEP group (10/30 vs. 14/22; *p*=0.030) in the 6^th^ month. In the 12^th^ month, this difference still had statistical significance. Besides, the ejaculatory volume was more in the modified group (1.5 ± 1.0 vs 1.0 ± 0.7, *p*=0.050 for 6^th^ month; 1.8 ± 0.6 vs 1.2 ± 0.8, *p*=0.003 for 12^th^ month). The frequency of urinary retention, permanent urinary incontinence, and urethral stricture did not show significant differences between the two groups.

## 4. Discussion

In our modified HoLEP technique, the mucous membrane of the bladder neck, the circular fibers of the internal urethral sphincter, and the urethral membrane between the 11 and 1 o'clock positions were preserved. Retrograde passage of semen is prevented by reflex closure of the bladder neck [14], and contraction of this sphincter prevents retrograde movement of semen into the bladder during ejaculation [10, 15]. This bladder neck closure mechanism is the etiology of RE after transurethral surgery for BPH [16]. Also, a wide array of studies confirms that bladder neck preservation done improves early return of urinary continence [17–20]. The preprostatic part of the urethra is about 1 cm long, extends from the base of the bladder to the prostate, and is associated with a circular cuff of smooth muscle fibers (the internal urethral sphincter) [10, 21]. Additionally, research has shown that there is a fibromuscular tissue called anterior lobe, which contains less glandular tissue than others in the abdomen of the prostate urethra [11]. Moreover, selective preservation of partial epithelia in the anterior wall of the urinary tract increases epithelialization of the urinary tract after surgical trauma and thus minimizes irritation of the surgical wound by urine and reduces scar formation [22, 23]. Beyond that, in our view, the urethral membrane, which was not cut off between the 11 and 1 o'clock positions, maintained the shape of prostatic fossa and helped to speed prostate contractions. In the current study, selective transurethral resection of the prostate, preserving the urethral membrane between the 11 and 1 o'clock positions, offers a more effective and safer alternative to TURP for small volume BPH patients [24].

The results showed that the occurrence rates of RE and UI were low at 33.33% and 1.03% in the modified group, respectively. The change of QoL score was better in the modified group than in the traditional group at 1^th^, 3^th^, 6^th^, and 12^th^ months after surgery. A large-scale multinational survey among approximately 14,000 men aged between 50 and 80 years, highlighted that the ejaculation function plays an important role in the QoL, even in aged men with symptomatic BPH [25]. Also, some articles reported that patients with RE had higher QoL scores [26–28] and agreed with our results. The change of maximum urinary flow rate was significantly lower in the modified group than in the traditional group. We think this is because the modified group preserved more detrusor tissue and urethral membrane than the traditional group, resulting in higher flow resistance. Although the decrease of the maximum urinary flow rate indicated the possibility of urethral stricture or obstruction, *Q*_max_ of two groups was still in the normal range. Besides, the frequency of postoperative urinary retention did not show significant differences between the two groups.

However, a recent study thought that bladder neck closure may not be important for maintaining antegrade ejaculation [7]. Kim et al. [7] listed two articles [29, 30], which have shown that retrograde ejaculation occurred in patients after retroperitoneal lymphadenectomy for testis tumor with a closed bladder neck. However, these two articles did not say RE would not occur when bladder neck was destroyed. Kim et al. also listed other three articles [31–33] in order to explain that patients could sustain orthostatic ejaculation after prostate sparing cystectomy and neobladder formation. However, there were some patients in all of these three articles who had retrograde ejaculation after surgery. In our opinion, when in the absence of comparison, we could not draw the conclusion that RE had no correction with the structure of bladder neck region. In our study, we selectively retained the mucous membrane of the bladder neck, the circular fibers of the internal urethral sphincter, and the urethral membrane between the 11 and 1 o'clock positions in the modified HOLEP group, but not in the traditional group. The results showed 33.33% rate of RE in the modified group, but 63.64% in the traditional group. It might be because of bladder neck closure mechanism and maintaining the shape of prostatic fossa.

Several studies have shown that HoLEP had a steeper learning curve than TURP, leading to hesitation to learn HoLEP among urologists. Placer et al. found that UI was seen in the early stages of the self-taught learning curve although the procedure remained effective [34]. Another study showed that an experience of more than 20 cases significantly helped a surgeon to decrease patients' post-HoLEP urinary incontinence [6]. Gong et al. [35] introduced a modified two-lobe enucleation technique of HoLEP that made work easier to perform and decreased operative time. In their study, the incidence of transient incontinence was 2% because of preserving the external sphincter. Recently, Miernik and Schoeb presented “3 horse shoe-like incision” HoLEP for bladder neck sparing which was easier to learn, but the incidence of postoperative UI and RE was not recorded and studied [36].

HoLEP continues to be a viable treatment option in BPH. Differences between surgical procedure designs may be factors that influence the therapeutic outcomes. Our study tried to introduce a new procedure for HoLEP to reduce the incidence of UI and RE. However, it had several limitations. Many patients discontinued the postoperative follow-ups, but the reasons for the dropouts were not investigated. It is possible that some of the dropouts developed severe complications, BPH recurrence, or sought treatment elsewhere. In addition, this study was a single-center study whose generalizability should be concerned. Next, it will be launched in the other institutions.

## 5. Conclusion

We found that the patients in the modified group suffered less postoperative urinary incontinence. Although UI had no significantly difference between two groups in the 12^th^ month, there was a declining trend in the figures of UI in the modified group. Moreover, RE and ejaculatory volume had significant difference in the 6^th^ and 12^th^ months. In the clinic, we also found that the patients in the modified group recovered urinary control quickly, winning acclaim and decreasing contradiction between patients and doctors.

## Figures and Tables

**Table 1:** Preoperative patients' characteristics.

Variables	Modified HoLEP	Traditional HoLEP	*p* value
Age (yr)	70.3 ± 7.1	71.9 ± 8.0	0.145^*∗*^
Prostate volume (cc)	71.3 ± 10.9	72.0 ± 11.1	0.661^*∗*^
PSA (ng/ml)	3.2 ± 1.51	3.3 ± 1.50	0.647^*∗*^
PVR (ml)	126.2 ± 101.9	139.9 ± 70.8	0.283^*∗*^
*Q*_max_ (ml/s)	7.0 ± 0.87	6.9 ± 0.91	0.438^*∗*^
IPSS	22.6 ± 2.02	22.2 ± 2.11	0.182^*∗*^
QoL score	4.7 ± 1.09	4.6 ± 1.07	0.523^*∗*^
Indwelling catheter	7.2% (7/97)	7.4% (7/94)	0.951^‡^
IIEF			
Erectile function	13.4 ± 6.7	13.0 ± 6.7	0.832^*∗*^
Orgasmic function	6.6 ± 3.6	5.5 ± 2.7	0.233^*∗*^
Intercourse satisfaction	4.9 ± 2.7	6.8 ± 4.4	0.060^*∗*^
Overall satisfaction	5.5 ± 3.7	4.6 ± 5.2	0.469^*∗*^

HoLEP = holmium laser enucleation of the prostate; PSA = prostate-specific antigen; PVR = postvoid residual urine volume; *Q*_max_ = maximum flow rate; IPSS = International Prostate Symptom Score; QoL = quality of life; IIEF = International Index of Erectile Function; ^*∗*^*t*-test; ^‡^Pearson's chi-square test.

**Table 2:** Perioperative data.

Variables	Modified HoLEP	Traditional HoLEP	*p* value
Operative time (min)	72.1 ± 20.3	70.1 ± 22.7	0.521^*∗*^
Enucleation time (min)	50.1 ± 15.1	49.2 ± 13.9	0.669^*∗*^
Morcellation time (min)	15.6 ± 8.8	15.2 ± 8.3	0.747^*∗*^
Transfusion rate, %	0	0	
Hospitalization (days)	3.5 ± 1.22	3.6 ± 1.47	0.609^*∗*^
Catheterization (days)	2.7 ± 0.6	2.8 ± 0.8	0.329^*∗*^
HGB levels drop (g/dl)	1.7 ± 1.1	1.9 ± 1.4	0.273^*∗*^

^*∗*^ *t*-test.

**Table 3:** Patient outcome scores over time.

Follow-up	1 month	3 months	6 months	12 months
No. of patients				
Modified	97	93	89	84
Traditional	94	91	86	82
PVR change (ml)				
Modified	−86.0 ± 57.1	−92.4 ± 55.0	−93.3 ± 49.6	−97.1 ± 58.5
Traditional	−102.0 ± 55.9	−107.9 ± 56.2	−108.6 ± 60.2	−111.4 ± 42.6
*p* value	0.052^*∗*^	0.060^*∗*^	0.068^*∗*^	0.074^*∗*^
IPSS score change				
Modified	−13.8 ± 3.07	−15.8 ± 3.56	−16.3 ± 2.76	−16.6 ± 2.10
Traditional	−13.7 ± 3.41	−16.0 ± 3.29	−16.4 ± 2.51	−16.8 ± 2.83
*p* value	0.831^*∗*^	0.693^*∗*^	0.803^*∗*^	0.605^*∗*^
QoL score change				
Modified	−2.2 ± 0.99	−3.1 ± 0.63	−3.2 ± 0.58	−3.4 ± 0.52
Traditional	−1.8 ± 0.76	−2.8 ± 0.77	−3.0 ± 0.60	−3.2 ± 0.53
*p* value	0.002^*∗*^	0.004^*∗*^	0.026^*∗*^	0.015^*∗*^
*Q*_max_ change				
Modified	+15.3 ± 2.43	+16.6 ± 2.88	+17.4 ± 3.19	+17.3 ± 3.28
Traditional	+16.7 ± 4.12	+17.4 ± 3.71	+18.4 ± 4.23	+18.1 ± 3.16
*p* value	0.005^*∗*^	0.428^*∗*^	0.079^*∗*^	0.163^*∗*^
IIFF score change				
Modified			−1.3 ± 0.8	+0.8 ± 0.4
Traditional			−1.4 ± 0.9	+0.7 ± 0.4
*p* value			0.146^*∗*^	0.109^*∗*^

PVR = postvoid residual urine volume; IPSS = International Prostate Symptom Score; QoL = quality of life; *Q*_max_ = maximum flow rate; IIEF = International Index of Erectile Function; ^*∗*^*t*-test.

**Table 4:** Incidence of late complications.

Complication	Modified	Traditional	*p* value
Early postoperative complications			
Transient urinary incontinence	1.03% (1/97)	8.51% (8/94)	0.036^†^
Acute urinary retention	1.03% (1/97)	0	1.000^†^
Late postoperative complications			
6-month postoperative retrograde ejaculation	33.33% (10/30)	63.64% (14/22)	0.030^‡^
6-month postoperative ejaculatory volume (ml)	1.5 ± 1.0	1.0 ± 0.7	0.050^*∗*^
12-month postoperative urinary incontinence	0 (0/84)	2.38% (2/82)	0.242^§^
12-month postoperative urethral stricture	3.57% (3/84)	1.21% (1/82)	0.630^†^
12-month postoperative retrograde ejaculation	13.33% (4/30)	50% (11/22)	0.034^‡^
12-month postoperative ejaculatory volume (ml)	1.8 ± 0.6	1.2 ± 0.8	0.003^*∗*^
12-month postoperative re-TURP	0 (0/84)	0 (0/82)	—

BPH = benign prostatic hyperplasia; TURP = transurethral resection of the prostate; ^†^continuity correction chi-square test; ^‡^Pearson's chi-square test; ^§^Fisher's exact test; ^*∗*^*t*-test.

## Data Availability

The supplementary material contains the original data of this study.

## Abbreviations

BPH: Benign prostatic hyperplasia
LUTS: Lower urinary tract symptoms
HoLEP: Holmium laser enucleation of the prostate
TURP: Transurethral resection of the prostate
PVP: Photovaporization of the prostate
RE: Retrograde ejaculation
UI: Urinary incontinence
IPSS: International Prostate Symptom Score
TRUS: Transrectal ultrasonography
*Q*_max_: Maximum urinary flow rate
PVR: Postvoid residual urine volume
QoL: Quality of life
IIEF: International Index of Erectile Function.

## Supplementary Materials

Supplementary Materials The supplementary file is the original data for the results of all patients. Sheet1: modified group. Sheet2: traditional group.
